# Plasma Levels of Amino Acids Related to Urea Cycle and Risk of Type 2 Diabetes Mellitus in Chinese Adults

**DOI:** 10.3389/fendo.2019.00050

**Published:** 2019-02-18

**Authors:** Yun-Feng Cao, Jing Li, Zhipeng Zhang, Jinnan Liu, Xiao-Yu Sun, Xiao-Fei Feng, Hui-Huan Luo, Wen Yang, Sai-Nan Li, Xilin Yang, Zhong-Ze Fang

**Affiliations:** ^1^Key Laboratory of Contraceptives and Devices Research (NPFPC), Shanghai Engineering Research Center of Reproductive Health Drug and Devices, Shanghai Institute of Planned Parenthood Research, Shanghai, China; ^2^Department of Epidemiology and Biostatistics, School of Public Health, Tianjin Medical University, Tianjin, China; ^3^Department of Surgery, Peking University Third Hospital, Beijing, China; ^4^Key Laboratory of Liaoning Tumor Clinical Metabolomics (KLLTCM), Jinzhou, Liaoning, China; ^5^Department of Toxicology and Sanitary Chemistry, School of Public Health, Tianjin Medical University, Tianjin, China; ^6^Tianjin Key Laboratory of Environment, Nutrition and Public Health, Tianjin, China

**Keywords:** type 2 diabetes, amino acid, urea cycle, metabolism, Chinese

## Abstract

**Objective:** This study aimed to test associations between type 2 diabetes mellitus (T2DM) and metabolites in urea cycle including arginine, citrulline and ornithine.

**Methods:**This study used a hospital-based cross-sectional study design. We retrieved medical notes of 401 in-patients with onset of T2DM within 2 years and 1,522 healthy subjects who attended annual physical examination. All cases were admitted to a tertiary care center in Jinzhou, China from May 2015 to August 2016. Binary logistic regression analyses were performed to obtain odds ratios (ORs) and 95% confidence intervals (CIs).

**Results:**Patients with T2DM had higher arginine, and lower ornithine than control subjects. Levels of citrulline were similar in two groups. Arginine was positively associated with T2DM (ORs: 1.20, 1.17–1.23) while ornithine was negatively associated with T2DM (OR: 0.89, 0.88–0.91). After adjustment for other amino acids and traditional risk factors, these associations were still significant and persistent for arginine and ornithine. The association between citrulline and T2DM was not significant. Their ratios of pairs of two amino acids were associated with increased risk of T2DM. After adjustment for other ratios of amino acids, effect size for T2DM remained significant. Further adjustment for traditional risk factors did not lead to large changes (ORs: 1.78, 1.20–2.65 for the ratio of arginine to ornithine; ORs: 1.59, 1.37–1.86 for the ratio of citrulline to ornithine, respectively) except the ratio of arginine to citrulline.

**Conclusions:** Plasma levels of amino acids related to urea cycle and their ratios of these amino-acids were associated with T2DM in Chinese adults.

## Introduction

Prevalence of diabetes has been continuing to soar in the world and the number of people with type 2 diabetes mellitus (T2DM) was estimated to hit 642 million by 2040 (1). T2DM is associated with serious complications (e.g., cardiovascular disease, kidney failure, and blindness) (2, 3) and contributes to an increased risk of cardiovascular mortality and morbidity. It poses a major burden on global economy and healthcare systems. Particularly, the burden of diabetes is high in developing countries because of rapid economic transformation, aging, urbanization, and lifestyle changes over a relatively short period and consequently, a more rapid increase in the prevalence of diabetes (4). Strong evidence indicates that T2DM can be prevented or delayed by modification of lifestyles (1, 5), which means that the burden of T2DM might be greatly reduced by prevention of diabetes in individuals at high-risk and early diagnosis or identification of undiagnosed patients with T2DM. During the last decades, advances in metabolomics have helped find many pathways or novel biomarkers involved in diabetes and its complications (6).

Metabolic disorders of carbohydrate, fat and protein follow diabetes after its diagnosis. These metabolic disturbances may occur well before onset or diagnosis of diabetes. Studies showed that abnormal protein metabolism was associated with insulin resistance (7) and oxidative stress (8). In patients with T2DM, the disturbance of protein metabolism is in a net catabolic state due to increased loss of nitrogen or negative nitrogen balance (i.e., amino acid catabolism is exaggerated and uptake of amino acids by tissues is reduced), even without protein restriction. Recently, studies reported that elevated levels of blood urea nitrogen were associated with increased risk of incident T2DM both in epidemiological studies (9) and experimental studies (10). Urea formation mainly occurs in the urea cycle in the liver, and the products and substrates of urinary metabolites include arginine, citrulline and ornithine. Animal data demonstrated significant differences of urinary metabolic profiles between high-fat-fed rats and their control rats (11, 12). Moreover, alterations of amino acids metabolism in urea cycle might be associated with insulin resistance (12). In the cycle, several enzymes involved in removal of nitrogen from amino acids are regulated by glucagon and insulin. These enzymes are also involved in synthesis of nitric oxide. In this regard, it is known that insulin has impacts on the activity of arginase (key enzyme in urea cycle) and nitric oxide synthase (NOS) (13, 14). Pietzner et al. (15) and Carracedo et al. (16) showed that amino acids involved in urea cycle were associated with inflammatory markers and oxidative stress. Given the connections between urea cycle disorder and many features of diabetes, we hypothesized that plasma levels of arginine, citrulline and ornithine were associated with the risk of T2DM.

Using a cross-sectional survey of 2,554 subjects in Jinzhou, China, we aimed to test the hypothesis that plasma levels of amino acids involved in the urea cycle were associated with the risk of T2DM.

## Materials and Methods

### Study Population and Settings

Liaoning Medical University First Affiliated Hospital (LMUFAH) is a comprehensive tertiary care hospital serving a population of 3.1 million in Jinzhou city, Liaoning province, China. The subjects of this study included in-patients with newly diagnosed T2DM (duration of diabetes ≤ 2 years) and healthy subjects who took part in a routine health check-up in LMUFAH. The details of the study subjects were described elsewhere (17). Briefly, the subjects who were selected into the study underwent the measurement of metabolism profile on a voluntary basis at LMUFAH between May 2015 and August 2016. The exclusion criteria were: (1) below 18 years old; (2) being a local resident in the hospital's catchment area for less than 6 months living prior to the study; (3) diabetes secondary to other diseases; and (4) mental diseases may bring trouble to complete health checks. The inclusion criteria included: (1) if being case group, patients with T2DM (duration ≤ 2 years) should be diagnosed by the 1999 World Health Organization's criteria (18) or treated with anti-diabetic medicines; and (2) if being control group, they did not have T2DM or other related diseases.

Based on the inclusion and exclusion criteria, we retrieved medical notes stored in a computer system to organize this hospital-based cross-sectional study. The retrieved data comprised anthropometric measurements, clinical laboratory assays, metabolism profile and other related data. Anthropometric data on height, weight and blood pressure were recorded. Clinical laboratory assays included glycated hemoglobin (HbA1c) (only available for patients with T2DM) and lipid profiles. Metabolomics assessment extracted data of amino acids involved in urea cycle, i.e., arginine, ornithine and citrulline. Additionally, drug use and diabetes complications, duration of diabetes were collected from all the subjects with T2DM. In total, we included data of 1,923 subjects (569 women and 1,354 men) in the analysis. Of them, 401 subjects had T2DM.

The protocol of the study was approved by the Ethics Committee for Clinical Research of LMUFAH and the informed consent was waived due to the nature of the retrospective study, which was in accordance with the Helsinki Declaration of 1964 and its later amendments.

### Data Collection and Definitions

Body weight, height and blood pressure were measured by specially trained doctors and nurses using standardized methods. When measuring weight and height, participants were required to take off shoes and heavy clothing. The measurements of weight and height were rounded to the nearest 0.1 kg and 0.5 cm, respectively. Body mass index (BMI) was calculated by dividing weight in kilograms by squared height in meters. According to the criteria recommended by the National Health Commission in China (19), BMI was divided into four categories: underweight (< 18.5 kg/m^2^), normal weight (18.5–23.9 kg/m^2^), obesity (24–27.9 kg/m^2^) and overweight (≥28 kg/m^2^). Blood pressure were measured after at least 10 min of rest in a sitting position.

Blood samples were collected from participants after an overnight fast of at least 8 h. Lipid profile was assayed, including high-density lipoprotein cholesterol (HDL-C), low-density lipoprotein cholesterol (LDL-C) and triglyceride (TG). Low HDL-C was defined as less than 1 mmol/L in men and 1.3 mmol/L in women (20). The metabolomic profiling including arginine, ornithine and citrulline was measured in all subjects.

Additionally, drug use and diabetes complications, duration of diabetes were collected from all the subjects with T2DM.

### Amino Acids Quantification

The metabolomics assessment method has been described previously in detail (21). Briefly, the metabolomics approach was based on mass spectrometry (MS) technology. We collected capillary whole blood after at least 8 h fasting which was stored as dried blood spot (DBS) and used in the assay of metabolomics. Metabolites in DBS were measured by direct infusion MS technology equipped with AB Sciex 4000 QTrap system (AB Sciex, Framingham, MA, USA). High-purity water and Acetonitrile from Thermo Fisher (Waltham, MA, USA) were used as diluting agent and mobile phase.1-Butanol and acetyl chloride from Sigma-Aldrich (St Louis, MO, USA) was used to derive samples. Isotope-labeled internal standard samples of 12 amino acids (NSK-A) were purchased from Cambridge Isotope Laboratories (Tewksbury, MA, USA) while standard samples of the amino acids were purchased from Chrom systems (Grafelfing, Germany).

### Data Analyses and Statistics

Statistical analyses were performed using Statistical Analysis System (Release 9.2) (SAS Institute Inc., Cary, USA). Data were expressed as mean ± SD (standard deviation) if normal distributions were not rejected or expressed as median with interquartile (IQR). Categorical data were expressed as numbers (percentages). Student's *t*-test or Mann Whitney *U* test was used in continuous data and Chi-squared test was used in categorical data to compare the differences between two groups. Binary logistic regression was conducted to obtain odds ratios (ORs) and their 95% confidence intervals (CIs) of arginine, ornithine, citrulline and the ratios of any two of them for the risk of T2DM in univariate and multivariate analyses. Confounding factors including age, gender, BMI, systolic blood pressure, HDL-C, LDL-C and triglyceride were adjusted in multivariable models.

Sensitivity analysis was also performed to check consistency of the results after exclusion of patients with any diabetic complications. A *p*-value in all results less than 0.05 was accepted as statistically significant.

## Results

### Characteristics of the Study Population

In this study, the mean age was 47.6 (SD: 14.1) years. Newly diagnosed patients with diabetes (duration ≤ 2 years) had the means of glycosylated hemoglobin (HbA1c) was 10.1% (SD: 2.4). About 12~14% of them had coronary heart disease and stroke, and 5~13% had diabetic retinopathy and diabetic nephropathy. Compared with control group, patients with T2DM were likely to be much older, lighter, shorter, and had lower concentrations of HDL-C and LDL-C, higher blood pressure and higher levels of triglyceride, but similar levels of BMI (Table 1).

**Table 1:** Clinical and biochemical characteristics of subjects according to occurrence of type 2 diabetes mellitus (T2DM).

**Variables**	**Non T2DM (1522)**	**T2DM (401)**	***P*-value**
	**Mean/number (SD or %)**	**Mean/number (SD or %)**	
Age	46.3 ± 13.7	52.5 ± 14.5	< 0.001
Duration of diabetes, years		5 (0–10)	
Male Gender	1131 (74.3%)	223 (55.6%)	< 0.001
Weight, kg	73.6 ± 13.5	71.2 ± 14.3	0.001
Height, cm	169.7 ± 8.0	167.0 ± 8.1	< 0.001
BMI, kg/m^2^	25.4 ± 3.5	25.4 ± 4.2	0.987
BMI < 18.5	23 (1.5%)	14 (3.5%)	
BMI ≥18.5 and < 24	504 (33.1%)	132 (32.9%)	
BMI ≥24 and < 28	653 (42.9%)	156 (38.9%)	
BMI ≥28	342 (22.5%)	99 (24.7%)	
SBP, mmHg	130.9 ± 17.2	136.2 ± 22.3	< 0.001
DBP, mmHg	81.0 ± 11.6	83.1 ± 12.6	0.002
HDL-C, mmol/L	1.55 ± 0.35	1.06 ± 0.35	< 0.001
Male (HDL-C < 1.0 mmol/L)	54 (3.6%)	89 (22.2%)	< 0.001
Female (HDL-C < 1.3 mmol/L)	40 (2.5%)	96 (23.9%)	
LDL-C, mmol/L	3.06 ± 0.70	2.93 ± 1.00	0.007
Triglyceride, mmol/L	1.51 (1.02–2.35)	1.81 (1.16–2.59)	0.013
Arg, μmol/L	4.47 (3.07–6.70)	9.74 (5.33–16.61)	< 0.001
Orn, μmol/L	53.07 (36.41)	17.01 (12.91–22.54)	< 0.001
Cit, μmol/L	19.07 (14.99–24.06)	18.5 (14.6–24.2)	0.284
Arg: Orn, %	7.76 (4.87–12.0)	59.41 (31.80–93.99)	< 0.001
Arg: Cit, %	24.01 (16.92–33.61)	55.29 (29.94–84.51)	< 0.001
Cit: Orn, %	32.00 (23.11–44.71)	110.69 (79.87–143.54)	< 0.001
HbA1C, %		10.1 ± 2.4	
**MACROVASCULAR COMPLICATIONS**			
Prior CHD		48 (12.0%)	
Prior stroke		57 (14.2%)	
**MICROVASCULAR COMPLICATIONS**			
Diabetic retinopathy		22 (5.5%)	
Diabetic nephropathy		53 (13.2%)	
**DRUG USE**			
Oral antidiabetic drugs		235 (58.6%)	
Insulin		287 (71.6%)	
Statins		141 (35.2%)	
Other lipid lowering drugs		11 (2.7%)	
ACEIs		34 (8.5%)	
ARBs		39 (9.7%)	
Other antihypertensive drugs		80 (20.0%)	

*BMI, body mass index; SBP/DBP, systolic/diastolic blood pressure; HDL-C, high density lipoprotein cholesterol; LDL-C, low density lipoprotein cholesterol; Arg, arginine; Orn, ornithine; Cit, citrulline; HbA1c, glycated hemoglobin; CHD, coronary heart disease; ACEIs, angiotensin-converting enzyme inhibitors; ARBs, angiotensin II receptor antagonists. Data are mean (SD), median (IQR), or n (%); P-values were derived from Chi-square test or Student t-test*.

For the metabolite profiles of amino acids in urea cycle, we observed significant differences in arginine and ornithine between the two groups. Compared with the control group, the serum concentrations of arginine was significantly higher and ornithine was markedly lower in patients with T2DM. Citrulline was similar between the two groups. In addition, the ratios of arginine to ornithine, arginine to citrulline and citrulline to ornithine were significantly higher in T2DM than in the control group (Table 1).

### Associations Between Amino Acids and T2DM

Arginine was positively associated with the risk of T2DM in univariate analyses. After further adjustment for each amino acid, the positive association was still significant and were strengthened for arginine (OR: 1.26, 95% CI:1.21–1.30) (Table 2). However, ornithine was inversely associated with the risk of T2DM. The multivariable-adjusted OR of ornithine for T2DM were 0.90 (95% CI 0.88–0.91). After adjustment for traditional risk factors, the effect sizes of amino acids remained almost unchanged (OR for arginine: 1.28, 1.21–1.35 and OR for ornithine: 0.91, 0.90–0.93, respectively). Citrulline was associated with the risks of T2DM (1.03, 1.01–1.06) after adjusting for arginine and ornithine. But further adjustment for confounders, the association was non-significant (Table 2).

**Table 2:** Odds ratio of amino acids and ratios of amino acids for the risk of type 2 diabetes mellitus.

	**Beta**	**OR**	**95%CI**	***P*-value**	**AUC**
**UNIVARIABLE MODEL 1**					
Arginine, μmol/L	0.18	1.20	1.17–1.23	< 0.0001	0.759
Ornithine, μmol/L	−0.11	0.89	0.88–0.91	< 0.0001	0.936
Citrulline, μmol/L	−0.002	1.00	0.98–1.01	0.790	0.517
Multivariable Model 1					0.954
Arginine, μmol/L	0.23	1.26	1.21–1.30	< 0.0001	
Ornithine, μmol/L	−0.11	0.90	0.88–0.91	< 0.0001	
Citrulline, μmol/L	0.03	1.03	1.01–1.06	0.0199	
Multivariable Model 2^*^					0.969
Arginine, μmol/L	0.25	1.28	1.21–1.35	< 0.0001	
Ornithine, μmol/L	−0.09	0.91	0.90–0.93	< 0.0001	
Citrulline, μmol/L	0.02	1.02	0.98–1.06	0.3889	
**UNIVARIABLE MODEL 2**					
Arginine:Ornithine, per 0.1	1.15	3.16	2.79–3.58	< 0.0001	0.944
Arginine:Citrulline, per 0.1	0.49	1.64	1.55–1.74	< 0.0001	0.789
Citrulline:Ornithine, per 0.1	0.67	1.96	1.83–2.09	< 0.0001	0.939
Multivariable Model 3					0.960
Arginine:Ornithine, per 0.1	0.36	1.44	1.08–1.92	0.0141	
Arginine:Citrulline, per 0.1	0.25	1.29	1.08–1.55	0.0060	
Citrulline:Ornithine, per 0.1	0.54	1.71	1.52–1.92	< 0.0001	
Multivariable Model 4^*^					0.982
Arginine:Ornithine, per 0.1	0.58	1.78	1.20–2.65	0.0044	
Arginine:Citrulline, per 0.1	0.16	1.17	0.91–1.51	0.2165	
Citrulline:Ornithine, per 0.1	0.47	1.59	1.37–1.86	< 0.0001	

*OR, odds ratio; CI, confidence interval; AUC, area under the curve*.

* *Adjusted for age, gender, body mass index, systolic blood pressure, high-density lipoprotein cholesterol, low-density lipoprotein cholesterol, and triglyceride*.

### Ratios of Amino Acids and the Risk of T2DM

We further analyzed the associations between ratios of amino acids in the urea cycle and T2DM. The ratios of arginine to ornithine, arginine to citrulline and citrulline to ornithine were all significantly associated with T2DM. After adjustment for other ratios of amino acids, the multivariable ORs per 0.1 of arginine to ornithine, arginine to citrulline, as well as citrulline to ornithine for T2DM among participants were 1.44 (95% CI 1.08–1.92), 1.29 (95% CI 1.08–1.55) and 1.71 (95% CI 1.52–1.92), respectively. Further adjustment for traditional risk factors did not lead to large changes and the associations remained significant except the association between ratio of arginine to citrulline and T2DM (Table 2).

### Sensitivity Analysis

After exclusion of patients with diabetic complications (*n* = 147), the effect sizes of arginine and ornithine, and their ratios for T2DM remained stable and significant in univariable and multivariable analyses (Table 3).

**Table 3:** Odds ratio of amino acids and ratios of amino acids for the risk of type 2 diabetes mellitus after exclusion of patients with diabetic complications.

	**Beta**	**OR**	**95%CI**	***P*-value**	**AUC**
**UNIVARIABLE MODEL 1**					
Arginine, μmol/L	0.18	1.20	1.17–1.23	< 0.0001	0.755
Ornithine, μmol/L	−0.12	0.89	0.88–0.90	< 0.0001	0.930
Citrulline, μmol/L	−0.02	0.98	0.96–1.00	0.0450	0.554
Multivariable Model 1					0.950
Arginine, μmol/L	0.23	1.25	1.20–1.31	< 0.0001	
Ornithine, μmol/L	−0.11	0.89	0.88–0.91	< 0.0001	
Citrulline, μmol/L	0.01	1.01	0.98–1.04	0.5689	
Multivariable Model 2^*^					0.965
Arginine, μmol/L	0.25	1.28	1.20–1.37	< 0.0001	
Ornithine, μmol/L	−0.11	0.90	0.88–0.92	< 0.0001	
Citrulline, μmol/L	0.002	1.00	0.95–1.06	0.9533	
**UNIVARIABLE MODEL 2**					
Arginine:Ornithine, per 0.1	1.10	3.00	2.63–3.43	< 0.0001	0.942
Arginine:Citrulline, per 0.1	0.51	1.66	1.56–1.77	< 0.0001	0.799
Citrulline:Ornithine, per 0.1	0.65	1.92	1.79–2.06	< 0.0001	0.932
Multivariable Model 3					0.955
Arginine:Ornithine, per 0.1	0.46	1.58	1.15–2.18	0.0054	
Arginine:Citrulline, per 0.1	0.19	1.21	0.98–1.50	0.0731	
Citrulline:Ornithine, per 0.1	0.48	1.62	1.42–1.84	< 0.0001	
Multivariable Model 4^*^					0.977
Arginine:Ornithine, per 0.1	0.59	1.80	1.19–2.73	0.0052	
Arginine:Citrulline, per 0.1	0.17	1.19	0.91–1.54	0.2057	
Citrulline:Ornithine, per 0.1	0.44	1.55	1.32–1.82	< 0.001	

*OR, odds ratio; CI, confidence interval; AUC, area under the curve*.

* *Adjusted for age, gender, body mass index, systolic blood pressure, high-density lipoprotein cholesterol, low-density lipoprotein cholesterol, and triglyceride*.

## Discussion

In this cross-sectional study, we found that the elevated levels of arginine but reduced levels of ornithine were associated with the increased odds of T2DM in Chinese. The ratios of arginine to ornithine and citrulline to ornithine were also associated with markedly increased risk of T2DM, associations being largely independent of traditional risk factors. Citrulline and the ratio of arginine to citrulline were not associated with T2DM.

As of disturbances of protein metabolism, patients with diabetes have a much higher rate of protein depletion but a lower rate of protein synthesis. In amino acid catabolism, most of the ammonia released is in the form of urea from a process of urea cycle of liver. It remains largely unknown whether amino acids in the urea cycle have any relationship with T2DM, especially in Chinese. Earlier experiment studies in mice (10, 22) showed that urea was associated with impaired beta cell function, elevated levels of reactive oxygen species production, reduced insulin sensitivity and glucose intolerance. Using a huge cohort of 1,337,452 subjects in USA, Xie et al. (9) found that elevated levels of blood urea nitrogen augmented the risk of T2DM.

In this connection, our study observed that increased levels of arginine but decreased level of ornithine, as well as their related ratios were associated with newly diagnosed T2DM. These observations suggested that abnormal urea cycle metabolism in the liver might derange as compared with the normal people. Our findings are supported by a study conducted in USA (23) showing that the level of arginine was higher in patients with diabetes than in control subjects, although some studies showed that the level of arginine was reduced in patients with T2DM (12, 24, 25). Given the close link between T2DM and urea, we speculate that elevated levels of arginine may increase urea synthesis, because arginine can activate N-acetylglutamate synthase (AGA) which further activates carbamoyl phosphate synthetase-I (CPS-I) to start urea cycle. On the other hand, arginine is involved in nitric oxide (NO) synthesis in citrulline-NO cycle (26). Enhancement of NOS activity could augment blood flow to insulin-sensitive tissues (13). NO is an endogenous AMPK activator and can indirectly represses lipogenesis or gluconeogenesis (27). Probably, our results suggest that there is a lower conversion of arginine to citrulline in diabetic patients and that NOS activity associated with arginine metabolism is impaired, leading to reduce NO bioactivity. The arguments are consistently with the previous studies (28). Tessari et al. (28) analyzed the trial of 8 patients with T2DM and 10 control subjects, and reported that NO synthesis was reduced due to a defective arginine conversion to NOx rather than substrate-limited.

Plasma ornithine concentration was markedly lower in patients with T2DM than the controls. In this regard, another previous study found that plasma levels of ornithine were inversely associated with inflammation marker (15). It can be inferred that lower levels of ornithine might be a feature of inflammatory conditions. Meanwhile, the decreased levels of ornithine might stem from increased depletion of ornithine. Experimental research demonstrated that expression of ornithine decarboxylase was up-regulated in very early stage of diabetes (29). Increased activity of ornithine decarboxylase might promote conversion of ornithine to putrescine. Nevertheless, it is still unknown that the over-degradation of ornithine to putrescine are due to diabetes itself or to insulin resistance.

Our study had several limitations worth noting. First, the major limitation of this study is cross-sectional design, no causal relationship between disorder of urea cycle metabolism and diabetes can be derived. It is noticed that protein metabolism disorder is one of the typical symptoms of T2DM. Therefore, it is possible that the changes of arginine, citrulline and ornithine are the results from diabetes and complications. In our sensitivity analysis, the effects of amino acids on T2DM were relatively stable and suggested that, at least in part, these associations were more likely a cause rather than a consequence of complication (i.e., diabetic nephropathy). Second, our subjects with T2DM were in-patients with T2DM and they may have more severe T2DM than those non-hospitalized patients with T2DM. Although we only included those patients with onset of T2DM within 2 years and made careful adjustment for disease severity such as onset age, blood pressure, lipid profile and diabetes complication, the plasma glucose effects and confounding effect of disease severity may not be completely removed. Further studies are needed to validate these findings in well-designed surveys or cohorts. Third, it is a pity that many important variables such as lifestyle factors and concentration of urea were not documented or measured, which limited us to further analyze alterations related with amino acids in urea cycle.

Our study has important implications for public health. The diabetes epidemic poses a major health and economy threat to the world including China. Nowadays, China has over 113.9 million adults with T2DM, and the extreme increase in the prevalence of diabetes and its complications are expected in the future. Strong evidence indicates that many cases of T2DM could be prevented by lifestyle interventions. Thus, it is critically important to develop an accurate model to identify individuals at high risk of developing T2DM in order to prevent its development in the future. However, a number of predictive models failed to accurately predict incident T2DM (30), even including genetic factors used in the predicting tools (31). Our study suggests that amino acids in urea cycle may be candidate markers for predicting T2DM in Chinese if these findings can be replicated in cohort studies, especially, in China.

In conclusion, our findings suggested that amino acids involved in urea cycling were associated with T2DM. Further cohort studies are warranted to replicate our findings to check whether the plasma levels of amino acids involved in urea cycle have predictive values for T2DM in the future.

## Author Contributions

XY and Z-ZF conceived and designed the study. JLi and Y-FC analyzed the data and wrote the first draft. ZZ participated in further revision after receiving the reviewers' comments. JLiu, X-FF, H-HL, WY and S-NL provided data collection. X-YS gave critical comments and contributed to the writing of this manuscript. All authors approved the final version of this manuscript. XY, Z-ZF, Y-FC, JLi and ZZ take full responsibility for the study as a whole, including the study design, access to data, and the decision to submit and publish the manuscript.

### Conflict of Interest Statement

The authors declare that the research was conducted in the absence of any commercial or financial relationships that could be construed as a potential conflict of interest.
